# Prophylactic Topical Antibiotics in Fracture Repair and Spinal Fusion

**DOI:** 10.1155/2021/1949877

**Published:** 2021-10-14

**Authors:** Eric K. Kim, Claire A. Donnelley, Madeline Tiee, Heather J. Roberts, Ericka Von Kaeppler, David Shearer, Saam Morshed

**Affiliations:** ^1^University of California San Francisco, School of Medicine, San Francisco, California, USA; ^2^Institute for Global Orthopaedics and Traumatology, Department of Orthopaedics, University of California, San Francisco, California, USA; ^3^University of California San Francisco, Department of Orthopaedic Surgery, San Francisco, California, USA; ^4^University of California San Francisco, Department of Epidemiology and Biostatistics, San Francisco, California, USA

## Abstract

**Introduction:**

The objective of this systematic review with meta-analysis is to determine whether prophylactic local antibiotics prevent surgical site infections (SSIs) in instrumented spinal fusions and traumatic fracture repair. A secondary objective is to investigate the effect of vancomycin, a common local antibiotic of choice, on the microbiology of SSIs.

**Methods:**

An electronic search of PubMed, EMBASE, and Web of Science databases and major orthopedic surgery conferences was conducted to identify studies that (1) were instrumented spinal fusions or fracture repair and (2) had a treatment group that received prophylactic local antibiotics. Both randomized controlled trials (RCTs) and comparative observational studies were included. Meta-analysis was performed separately for randomized and nonrandomized studies with subgroup analysis by study design and antibiotic.

**Results:**

Our review includes 44 articles (30 instrumented spinal fusions and 14 fracture repairs). Intrawound antibiotics significantly decreased the risk of developing SSIs in RCTs of fracture repair (RR 0.61, 95% CI: 0.40–0.93, *I*^2^ = 32.5%) but not RCTs of instrumented spinal fusion. Among observational studies, topical antibiotics significantly reduced the risk of SSIs in instrumented spinal fusions (OR 0.34, 95% CI: 0.27–0.43, *I*^2^ = 52.4%) and in fracture repair (OR 0.49, 95% CI: 0.37–0.65, *I*^2^ = 43.8%). Vancomycin powder decreased the risk of Gram-positive SSIs (OR 0.37, 95% CI: 0.27–0.51, *I*^2^ = 0.0%) and had no effect on Gram-negative SSIs (OR 0.95, 95% CI: 0.62–1.44, *I*^2^ = 0.0%).

**Conclusions:**

Prophylactic intrawound antibiotic administration decreases the risk of SSIs in fracture surgical fixation in randomized studies. Therapeutic efficacy in instrumented spinal fusion was seen in only nonrandomized studies. Vancomycin appears to be an effective agent against Gram-positive pathogens. There is no evidence that local vancomycin powder is associated with an increased risk for Gram-negative infection.

## 1. Introduction

Surgical site infections (SSIs) are a significant source of morbidity and cost for patients undergoing orthopedic procedures. SSIs are challenging to treat because of the potential formation of a bacterial biofilm, an extracellular matrix that can attach to implants and protect pathogens from host immunity and systemic antibiotics [1]. Both instrumented spinal procedures and fracture surgeries have in common the use of metallic hardware, and each suffer from a nontrivial rate of SSIs, ranging from 9.4% of noninstrumented spinal trauma cases [2] to over 30% in lower extremity fracture cases [3, 4]. SSIs lead to delayed healing, nonunion, irreversible loss of function, or amputation of the infected limb [3, 4].

The current standard of care for SSI prevention is systemic antibiotics [5, 6]. However, parenterally administered antibiotics have the disadvantages of delivering a reduced concentration of antibiotics to the targeted site, failing to reach poorly vascularized tissues, and potentially causing systemic toxicity. Alternatively, locally delivered antibiotics can achieve a high local concentration with low systemic levels, thereby avoiding dangerous side effects such as nephrotoxicity or ototoxicity [1].

Despite these strengths associated with local antibiotic therapy, there are also concerns. One is that a high concentration of antibiotics can potentially inhibit new bone formation [7]. Another concern is the development of antibiotic resistance [8] or the emergence of pathogens not covered under the narrow spectrum of commonly used antibiotics such as vancomycin [9–11]. Additionally, prophylaxis with local antibiotics is an off-label usage and may cause unforeseen adverse events.

Previous systematic reviews have examined the effect of intrawound antibiotics in either instrumented spinal procedures or open limb fractures [9, 12–16]. Because both fields share a high risk of infection and the objective of achieving bony union, which may be affected by local antibiotics, we believe there is value in reporting pooled outcomes of local antibiotics comprehensively from both specialties. The outcomes of local antibiotics in extremity fracture treatment may therefore be generalizable to spinal fusion surgery and vice versa.

Therefore, the aim of this systematic review is to assess the efficacy of locally administered antibiotics in preventing SSIs in instrumented spinal procedures and fracture surgeries and to investigate the effect of local vancomycin powder on SSIs caused by Gram-negative organisms.

## 2. Materials and Methods

### 2.1. Search Strategy

We searched the PubMed, EMBASE, and Web of Science databases for our systematic review and meta-analysis. Keywords and phrases that guided our search strategy included “intrawound OR local” and “fracture OR fusion” and “prophylaxis OR prevent.” The following MeSH terms were developed from key articles and used on PubMed: “anti-infective agents, local” and “antibiotic prophylaxis” (full search strategy in Appendix A). All relevant abstracts presented at major orthopedic conferences (Orthopaedic Trauma Association Conference, American Academy of Orthopaedic Surgeons Conference, North American Spine Society, Scoliosis Research Society) and available on the conference databases were included. Two additional articles were identified from the bibliographies of included articles and relevant review papers. Our initial search was performed in 2019; a secondary search was performed in 2021 to identify updated, relevant articles.

### 2.2. Eligibility Criteria

Criteria for inclusion in this systemic review were studies that (1) included patients undergoing acute fracture repair or spinal fusion with instrumentation, (2) had a treatment group that received prophylactic local antibiotics, and (3) reported SSI as a primary outcome. We wanted to assess local antibiotic prophylaxis in instrumented procedures meant to achieve bone healing in adult studies.

We excluded studies of only pediatric patients. We included studies whose patient age range spanned children and adults because the age means with standard deviations of these articles indicated that the majority of the patients were adults. Studies of craniofacial surgeries were excluded because of the unique bacterial flora and vasculature of this anatomic region [17]. We excluded case series and studies without a control group. Studies of spinal decompression procedures without fusion (e.g., laminectomy) were excluded. For articles that investigated both decompression and/or instrumented procedures, only the infection results of instrumented cases were included. Studies in which the treatment group received other experimental therapies (e.g., antiseptics) in addition to local antibiotics were also excluded. Furthermore, there were some groups that published multiple studies on the same patient cohort, either at different time points during one collection period or with different subsets of the same data. In such cases, only the article with the greatest sample size was included. Finally, any articles that did not report patient data were excluded, such as narrative reviews, pharmacokinetic studies, and articles on novel antibiotic delivery systems. Any conflicts were resolved through discussion and consensus.

### 2.3. Study Identification and Data Extraction

**Figure 1:**
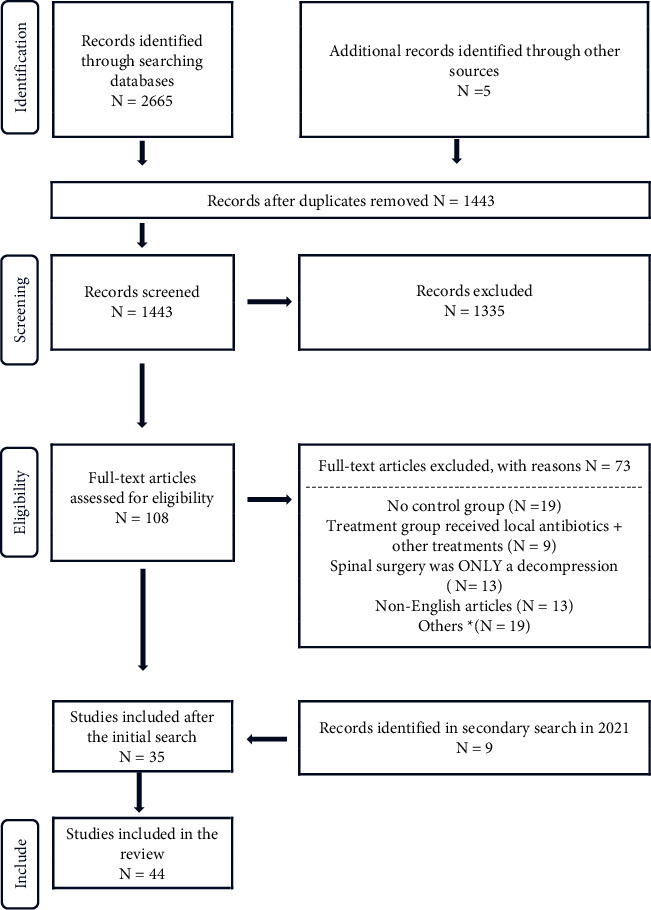
Literature search flowchart for Preferred Reporting Items for Systematic Reviews and Meta-Analyses (PRISMA). ^*∗*^ Excluded for the following reasons: no patient data, pediatric study, no local antibiotic usage, repeat study, animal study, not accessible, and includes surgeries that are not instrumented spinal fusion or fracture surgeries.

Two authors (EK and MT) individually conducted a title and abstract screening with DistillerSR (Evidence Partners, Ottawa, Canada). The full text articles of selected studies were separately assessed by two authors (EK and CD). The data were extracted independently by two authors (EK and CD), including an assessment of patient population, local antibiotic of choice, sample size, method of controlling for bias, number of infections, and the culture results, if available. The Preferred Reporting Items for Systematic Reviews and Meta-Analyses (PRISMA) flow diagram details the number of articles retrieved and excluded at each stage of the review (Figure 1).

### 2.4. Meta-Analysis and Subgroup Analysis

**Figure 2:**
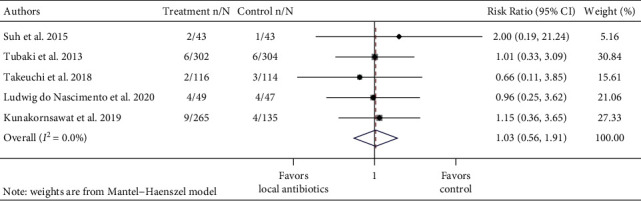
Forest plot of infection data of 5 instrumented spinal fusion randomized controlled trials. Treatment n/N: number of infections in the treatment group/total number of patients in the treatment group. Control n/N: number of infections in the control group/total number of patients in the control group.

**Figure 3:**
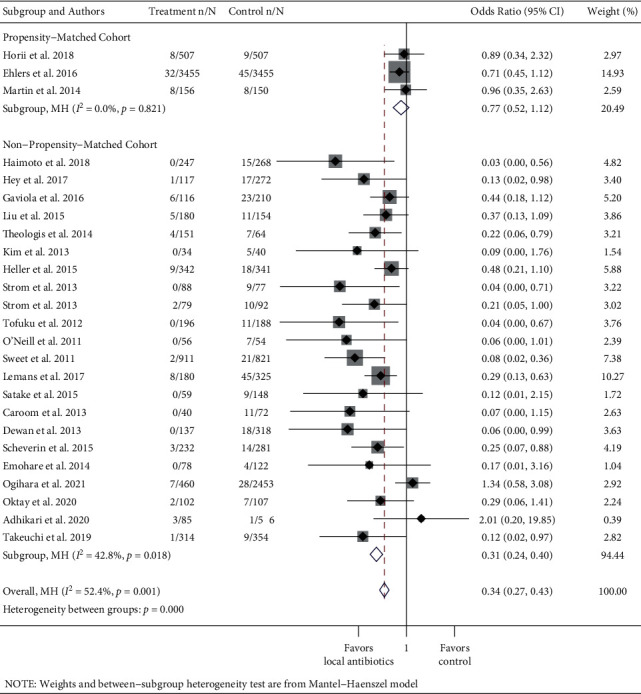
Forest plot of observational instrumented spinal fusion studies. Treatment n/N: number of infections in the treatment group/total number of patients in the treatment group. Control n/N: number of infections in the control group/total number of patients in the control group.

**Figure 4:**
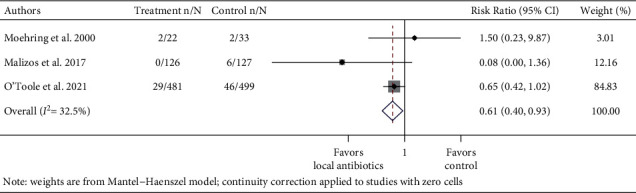
Forest plot of infection data of 3 fracture randomized controlled trials. Treatment n/N: number of infections in the treatment group/total number of patients in the treatment group. Control n/N: number of infections in the control group/total number of patients in the control group.

**Figure 5:**
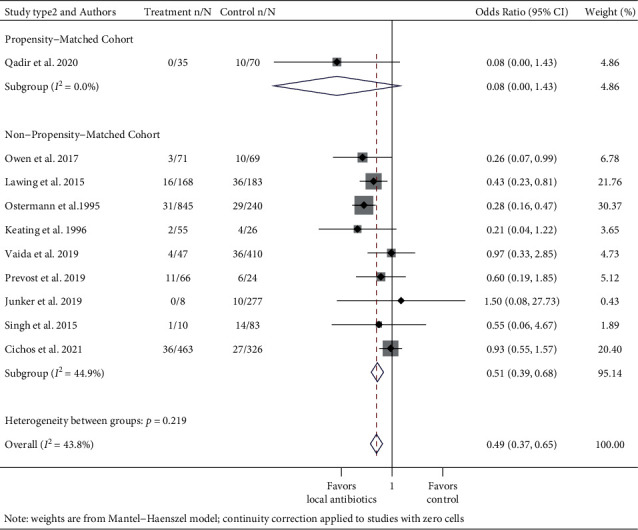
Forest plot of observational fracture studies. Treatment n/N: number of infections in the treatment group/total number of patients in the treatment group. Control n/N: number of infections in the control group/total number of patients in the control group.

To study whether prophylactic local antibiotics reduce the risk of SSIs, separate meta-analyses were performed for the instrumented spinal procedure and fracture repair. Further, RCTs and observational studies were pooled separately, with subgroups of study design among nonrandomized studies (propensity-matched cohort study or nonpropensity-matched cohort study) (Figures 2–5 ). Risk ratios (RRs) and odds ratios (ORs) were calculated in the meta-analyses of RCTs and observational studies, respectively.

**Figure 6:**
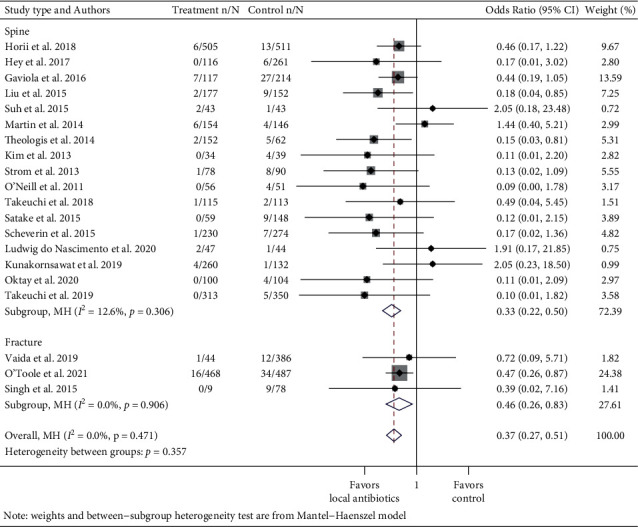
Forest plot of Gram-positive infection data of studies that used vancomycin with subgroup analysis for spine and fracture cases. Treatment n/N: number of infections in the treatment group/total number of patients in the treatment group. Control n/N: number of infections in the control group/total number of patients in the control group.

**Figure 7:**
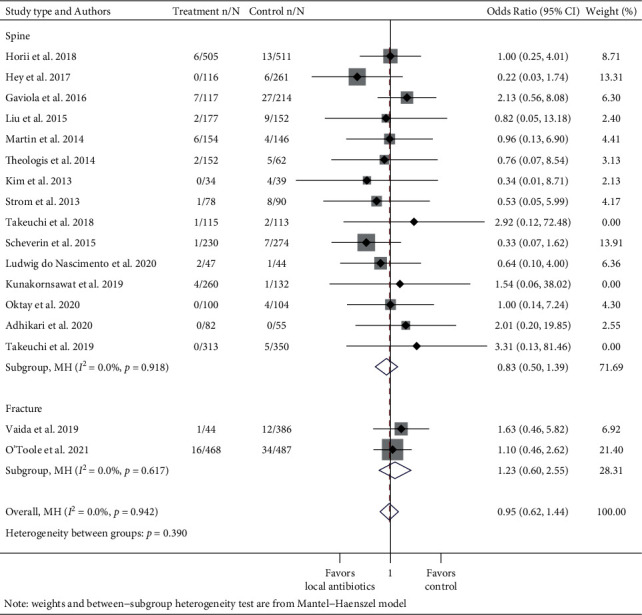
Forest plot of Gram-negative infection data of studies that used vancomycin with subgroup analysis for spine and fracture cases. Treatment n/N: number of infections in the treatment group/total number of patients in the treatment group. Control n/N: number of infections in the control group/total number of patients in the control group.

To evaluate the effect of vancomycin on Gram-positive and Gram-negative infections, data were pooled from articles that studied the use of vancomycin powder and reported SSI culture results. These data were grouped into meta-analyses of Gram-positive vs. Gram-negative organisms with subgroup analysis for spine and fracture studies (Figures 6 and 7). Negative or mixed polymicrobial results (both Gram-positive and Gram-negative pathogens cultured in the same infection) were omitted. When multiple organisms that were either all Gram-positive or all Gram-negative were cultured in one SSI, they were counted as one. Studies that provided bacterial data for only a portion of the SSIs were excluded from this subgroup analysis.

### 2.5. Statistical Analysis

STATA 16 software (Statacorp, College Station, TX) was used to conduct random-effects meta-analyses using the admetan command, which is built on the Mantel and Haenszel model to develop RRs and ORs for binary and continuous data [18, 19]. The heterogeneity of the included studies was quantified with the *I*^2^ statistic.

## 3. Results

### 3.1. Spinal Instrumentation

Thirty spinal instrumentation studies were included, with five RCTs, two prospective observational studies, and 23 retrospective studies (Table 1) [20–49]. A total of 17,756 patients were included. Among the nonrandomized studies, three studies were propensity-matched [23, 29, 35]. There was a wide variety in the type of instrumented procedure performed, reflecting a range of diagnoses across studies. Twenty-two of the 30 studies exclusively included fusion cases. The treatment group in 29 of 30 studies received vancomycin with varying dosages (0.5–2 g) and methods of application. The study that did not report the use of vancomycin did not specify either the antibiotic type or the dosage [23]. The primary outcome of included studies was SSI.

The pooled RR of infection in the treatment group compared to the control group across five spinal instrumentation RCT studies was 1.03 (95% CI: 0.56–1.91, *I*^2^ = 0.0%) (Figure 2). The pooled odds ratio (OR) of infection in the treatment group compared to the control group across all 25 spinal instrumentation observational studies was 0.34 (95% CI: 0.27–0.43, *I*^2^ = 52.4% (Figure 3). Subgroup analyses by the study type (propensity-matched cohort or non-propensity-matched cohort) among observational studies were performed. The pooled OR of the three propensity-matched cohort studies was 0.77 (95% CI: 0.52–1.12, *I*^2^ = 0.0). The remaining 22 studies had a pooled OR of 0.24 (95% CI: 0.17–0.32, *I*^2^ = 42.8%).

### 3.2. Fracture Repair

Fourteen fracture studies were included, with three RCTs, two prospective observational studies, and nine retrospective studies (Table 2) [50–63]. A total of 4,635 patients were included. Similar to the spine studies, the fracture studies reported SSI as the primary outcome. There was considerable clinical heterogeneity among the studies, such as fracture location, antibiotic type, and definition of SSI. Data from Bibbo and Patel [50] did not contribute to the meta-analysis because both the treatment and control groups had a 0% SSI incidence.

The pooled RR of infection in the treatment group in the three RCTs was 0.61 (95% CI: 0.40–0.93, *I*^2^ = 32.5%) (Figure 4). Subgroup analyses by the study type (propensity-matched cohort or non-propensity-matched cohort studies) among observational studies were also performed. Because there was only one propensity-matched cohort study, it was not pooled. The pooled odds ratio (OR) of infection in the treatment group compared to the control group across 10 observational fracture studies was 0.49 (95% CI: 0.37–0.65 *I*^2^ = 43.8%) (Figure 5). In the subgroup analysis by study type, the pooled OR for nine non-propensity matched cohort studies was 0.51 (95% CI: 0.39–0.68, *I*^2^ = 44.9%).

Because the fracture studies used a variety of antibiotics, subgroup analysis by antibiotic type was conducted, which pooled studies of all designs together (Appendix B). For the five vancomycin studies, the pooled OR was 0.71 (95% CI: 0.51–0.98, *I*^2^ = 0.0%). The pooled OR for the three tobramycin studies was 0.31 (95% CI: 0.19–0.50, *I*^2^ = 27.4%). Two studies used both vancomycin and tobramycin and showed a pooled OR of 0.41 (95% CI: 0.18–0.94, *I*^2^ = 0.0%). The study by Bibbo and Patel [50] was not included because both the treatment and control groups had a 0% SSI incidence. Three remaining studies [52, 54, 55] each had a unique antibiotic regimen and were not pooled with other studies.

### 3.3. Microbiology in Instrumented Spinal Procedures and Fracture Surgeries That Used Vancomycin

To address the concern that the use of local vancomycin powder can affect the incidence of Gram-negative infection, two meta-analyses spanning 20 studies that studied local vancomycin and reported culture data were conducted. The pooled OR for an infection caused by Gram-positive bacteria in the vancomycin group compared to the control group was 0.37 (95% CI: 0.27–0.51, *I*^2^ = 0.0%) (Figure 6). Subgroup analysis by spine and fracture cases revealed an OR of 0.33 (95% CI: 0.22–0.50, *I*^2^ = 12.6%) in instrumented spinal procedures and OR of 0.46 (95% CI: 0.26–0.83, *I*^2^ = 0.0%) in fracture repairs. The pooled OR for Gram-negative infection in the vancomycin group was 0.95 (95% CI: 0.62–1.44, *I*^2^ = 0.0%) (Figure 7). The subgroup analysis showed that the OR was 0.83 (95% CI: 0.50–1.39, *I*^2^ = 0.0%) for spinal instrumentation and 1.23 (95% CI: 0.60–2.55, *I*^2^ = 0.0%) for fracture surgeries. Three studies were excluded in the meta-analysis of Gram-negative infections because both control and treatment groups had no Gram-negative SSIs. Subgroup analyses by study type showed a similar trend towards greater effect size among nonrandomized studies.

## 4. Discussion

We performed a meta-analysis of 44 studies evaluating the effect of locally administered antibiotics on rates of infection after instrumented spine and fracture surgeries. Notable findings include a significant reduction in the pooled incidence of infection in both patient populations, but this effect was weaker or absent with more rigorous study designs. The pooled effect of vancomycin was significant for the reduction in Gram-positive infection and did not show any association with Gram-negative infection compared to no local antibiotics.

### 4.1. Spinal Instrumentation

Previous systematic reviews have demonstrated the benefit of local antibiotics and antiseptic prophylaxis. Dodson et al. [9] pooled 21 studies (2 RCTs and 19 observational studies) and found that prophylactic vancomycin powder significantly reduced the risk of developing SSIs in spinal surgeries (RR 0.55, 95% CI: 0.45–0.67, *p*=0.0001). Similarly, Lemans et al. [12] pooled 20 studies (2 RCTs and 18 observational studies) and showed that using preventive intrawound antibiotics and antiseptics also decreased the risk of deep SSIs in instrumented surgeries (RR 0.26, 95% CI: 0.17–0.51, *p* < 0.0001).

Surgical procedures with instrumentation have a higher risk of biofilm formation [1]. Therefore, our study focused exclusively on instrumented procedures in adult patient populations and yielded a result consistent with other systematic reviews [9, 12]. Neither the meta-analysis of the five RCTs nor the meta-analysis of the three propensity-matched cohort studies showed the same significant reduction that occurred with the pooling of cohort studies. Many observational studies used a “before-and-after” study design that is prone to confounding bias, which may explain the greater effect size observed in non-propensity-matched studies [64]. The blinded RCT remains the methodological gold standard for proving the efficacy of therapeutic intervention; it is important that any future observational studies incorporate design and analytical methods to control for bias, such as propensity score adjustment [65].

### 4.2. Fracture Repair

Previous systematic reviews found that intrawound antibiotics in open fractures reduced the risk of SSIs. Craig et al. [16] evaluated the role of local antibiotic prophylaxis in open tibia fractures treated with intramedullary nails in their meta-analysis of seven articles (one RCT and six observational studies). For patients with Gustilo–Anderson (GA) type III fractures, those who received only parenteral antibiotics had an infection rate of 14.4% (95% CI: 10.5%–18.5%). In comparison, those who received local prophylactic antibiotics had an infection rate of 2.4% (95% CI: 0.0–9.4), with an OR of 0.17. A meta-analysis of eight articles (one RCT and seven observational studies) by Morgenstern et al. [15] showed a similar significant reduction in infection risk in open fractures (OR = 0.30, 95% CI: 0.22–0.40).

Our meta-analysis showed a pooled benefit of prophylactic intrawound antibiotics in both randomized and nonrandomized studies. Similar to our analysis of spine instrumentation studies, bias reduction from randomization revealed that the magnitude of the effect is likely to be smaller than previously thought yet trending towards a protective effect. Subgroup analysis by antibiotic type showed that local vancomycin reduced SSI in fracture repair, which is in line with the established coverage pattern of vancomycin and common infectious organisms. Larger studies and trials that combine vancomycin with agents with Gram-negative coverage may be required to achieve the precision and possible added magnitude of effect to prove the impact of local antibiotics for the prevention of SSI in this population.

### 4.3. Microbiology with the Use of Vancomycin Powder

Our meta-analyses assessed the microbiology of SSIs by pooling culture data from studies that used vancomycin powder. We specifically addressed vancomycin because of its widespread use and concern that its selective coverage of Gram-positive pathogens may increase the incidence of Gram-negative infection [1]. In a study of 2802 patients undergoing spinal surgery, Chotai et al. [11] observed a lower incidence of deep SSIs in the vancomycin group. However, there was a higher percentage of SSI caused by Gram-negative organisms in the vancomycin group than in the control group (28% vs. 12.5%).

We demonstrated that vancomycin reduces Gram-positive infection and has no effect on Gram-negative infection in both instrumented spinal procedures and fracture surgeries. The effectiveness of vancomycin against Gram-positive pathogens is consistent with its established antibacterial spectrum covering some of the most commonly cultured organisms in SSI of both instrumented spinal procedures and fractures, including *Staphylococcus aureus* [8, 10, 11]. Furthermore, our results are reassuring to orthopedic surgeons who are apprehensive about the potential for vancomycin to increase the incidence of Gram-negative infections.

### 4.4. Adverse Events

No studies reported any adverse events attributable to local antibiotics. The majority of the included studies had a single sentence denying side effects. Some studies explicitly reported that intrawound antibiotics did not impact the rates of nonunion, addressing the concern that topical antibiotics can impede bone healing [7, 31, 42, 50, 54, 57]. It is important to note that most of the included studies were not powered to detect differences in pseudarthrosis.

## 5. Limitations

The primary limitation of this study is the pooling of cohort studies in the meta-analysis. We intentionally included both RCTs and observational studies because there are very few RCTs that investigate the prophylactic effect of intrawound antibiotics, but the RCTs and nonrandomized studies were analyzed separately. The majority of the current evidence is from observational studies. A high degree of heterogeneity in study design, outcome assessment, treatment protocols, and definition of SSI existed among the included studies. There was a hierarchy of study designs among observational studies across which differences in effect were identified.

## 6. Conclusion

Prophylactic topical antibiotics are associated with decreased risk of surgical site infection after both instrumented spine and fracture surgeries in much of the published literature on the topic. Although the effect is weak or absent in more rigorous study designs in the instrumented spinal fusion literature, pooling of fracture repair RCTs revealed that intrawound antibiotics significantly reduced SSIs. There is no evidence to suggest a higher incidence of Gram-negative infection or other adverse events among patients treated with local vancomycin, irrespective of study quality. These results do not support the use of local antibiotics in patients undergoing spinal fusion but suggest therapeutic efficacy in patients undergoing fracture repair.

## Figures and Tables

**Table 1:** Summary of infection rates and methodology of studies of instrumented spinal procedure.

Authors/year	Study design/method of controlling for bias	No. of pts	Age range of pts	Included spinal diagnoses/procedures	Intervention	Wound infection rates in treatment group	Wound infection rates in control group
Adhikari et al. 2020 [20]^‡^	RC/NR	141	Adults	Deformity, degenerative, trauma, neoplastic/posterior instrumented fusion	Vancomycin powder 1 g	3.53% (3/85)	1.79% (1/56)

Caroom et al. 2013 [21]^*∗*^	RC/NR	112	NR	Cervical spondylotic myelopathy/posterior instrumented fusion	Vancomycin powder 1 g	0% (0/40)	15.28% (11/72)

Dewan et al. 2013 [22]^a,^^*∗*^	RC/NR	455	NR	Degenerative/posterior spinal fusion	Vancomycin powder 1 g	0% (0/137)	5.66% (18/318)

Ehlers et al. 2016 [23]^b^	PC/propensity score matching	6910	NR	Instrumented cervical or lumbar fusion	Intrawound antibiotics (type and dose NR)	0.93% (32/3455)	1.30% (45/3455)

Emohare et al. 2014 [24]^c^	RC/multivariate analysis, pseudo-randomization by surgeon^d^	200	NR	Degenerative/posterior instrumented thoracic, thoracolumbar, lumbar fusion	Vancomycin powder 1 g	0% (0/78)	3.28% (4/122)

Gaviola et al. 2016 [25]	RC/multivariate analysis	326	40–71	Instrumented multilevel fusion	Vancomycin powder 2 g	5.17% (6/116)	11.0% (23/210)

Haimoto et al. 2018 [26]^*∗*^	RC/NR	515	18 and above	Posterior instrumented cervical, thoracic, lumbar fusion	Vancomycin powder 1 g	0% (0/247)	5.60% (15/268)

Heller et al. 2015 [27]^‡^	RC/NR	683	NR	Degenerative, deformity, neoplastic, others/posterior instrumented fusion	Vancomycin powder 0.5–2 g	2.63% (9/342)	5.28% (18/341)

Hey et al. 2017 [28]^*∗*^	RC/multivariate analysis, pseudo-randomization	389	11–85	Degenerative, trauma, neoplastic/open instrumentation	Vancomycin powder 1 g	0.85% (1/117, 1 deep)	6.25% (17/272, 10 deep, 7 superficial)

Horii et al. 2018 [29]^b^	RC/propensity score matching	1014	15 and above	Degenerative, deformity, trauma, neoplastics/posterior instrumentation	Vancomycin powder 1–2 g	1.58% (8/507)	1.78% (9/507)

Kim et al. 2013 [30]^*∗*^	RC/logistic regression, multivariate analysis, and cox regression	74	NR	Spinal instability/posterior instrumented fusion	Vancomycin powder 1 g	0% (0/34)	12.5% (5/40, 3 deep, 2 superficial)

Kunakornsawat et al. 2019 [31]^‡^	RCT/randomizations	400	11–82	Trauma, degenerative, congenital, neoplastic, infectious/posterior instrumented thoracic or lumbosacral fusions	Vancomycin powder 1–2 g	3.40% (9/265)	2.96% (4/135)

Lemans et al. 2017 [32]^*∗*^	RC/NR	505	Adults	Open posterior instrumentation	Vancomycin powder 1–2 g	4.44% (8/180, 5 deep, 3 superficial)	13.85% (45/325, 31 deep, 14 superficial)

Liu et al. 2015 [33]^‡^	RC/NR	334	53.5–74	Deformity, degenerative, neoplastic/posterior instrumentation	Vancomycin powder 0.5–2 g	2.78% (5/180)	7.14% (11/154)

Ludwig do nascimento et al. 2020 [34]	RCT/randomization, double blinding	96	17–74	Degenerative, trauma/thoracolumbar spine arthrodesis	20 ml of saline with 2 g of diluted vancomycin	8.16% (4/49)	8.51% (4/47)

Martin et al. 2014 [35]^b,‡^	RC/logistic regression, propensity score matching	306	18 and above	Deformity/posterior instrumented fusion	Vancomycin powder 2 g	5.12% (8/156)	5.33% (8/150)
Ogihara et al. 2021 [36]^‡^	RC/multivariable analysis	2913	18–93	Degenerative/posterior instrumented fusion in the thoracic/lumbar spines	Vancomycin powder	1.52% (7/460)	1.14% (28/2453)

Oktay et al. 2020 [37]^*∗*^	RC/NR	209	14–90	Degenerative, trauma, neoplastic, revision/posterior instrumentation	Vancomycin powder 1 g	1.96% (2/102, 1 deep, 1 superficial)	6.54% (7/107, 4 deep, 3 superficial)

O'Neill et al. 2011 [38]^*∗*^	RC/pseudo-randomization	110	18 and above	Trauma/posterior instrumented fusion	Vancomycin powder 1 g	0% (0/56)	12.96% (7/54, 5 deep, 2 superficial)

Satake et al. 2015 [39]^‡,^^*∗*^	PC/NR	207	Not given	Open posterior instrumented thoracic, lumbar fusion	Vancomycin powder with fibrin glue (dosage NR)	0% (0/59)	6.08% (9/148)

Scheverin et al. 2015 [40]^*∗*^	RC/pseudo-randomization	513	18–78	Degenerative/posterior instrumented lumbar fusion	Vancomycin powder 1 g mixed with bone graft	1.29% (3/232)	4.98% (14/281)

Strom et al. 2013 [41]^*∗*^	RC/NR	171	Adult patients	Degenerative, infectious, neoplastic, trauma/posterior cervical instrumented fusion	Vancomycin powder 1 g	2.53% (2/79)	10.87% (10/92)

Strom et al. 2013 [42]^*∗*^	RC/stratification	165	NRs	Degenerative, infectious, neoplastic, trauma/lumbar laminectomy and posterior instrumented fusion	Vancomycin powder 1 g	0% (0/88)	11.69% (9/77)

Suh et al. 2015 [43]	RCT/NR	86	23–83	Degenerative/posterior instrumented lumbar fusion	Vancomycin powder 2 g	4.65% (2/43)	2.33% (1/43)

Sweet et al. 2011 [44]^‡,^^*∗*^	RC/NR	1732	12–86	Posterior instrumented thoracolumbar fusions	Vancomycin powder 2 g	0.22% (2/911)	2.56% (21/821)

Takeuchi et al. 2018 [45]^d^	RCT/randomization, blinding	230	NR	Deformity, degenerative, trauma/thoracic, lumbar fusion	Vancomycin powder 1 g	1.72% (2/116, 1 deep, 1 superficial)	2.63% (3/114, 1 deep, 2 superficial)

Takeuchi et al. 2019 [46]^*∗*^	RC/NR	668	16–89	Degenerative, fracture/posterior spinal instrumentation	Vancomycin powder 1 g	0.32% (1/314)	2.54% (9/354)

Theologis et al. 2014 [47]^*∗*^	RC/NR	215	18–88	Deformity/fusion greater than 3 levels	Vancomycin powder 2 g	2.65% (4/151)	10.93% (7/64)

Tofuku et al. 2012 [48]^‡,^^*∗*^	RC/NR	384	7–89	Degenerative, neoplastic, trauma, infectious/spinal instrumentation	0.5 g Vancomycin-impregnated fibrin sealant	0% (0/196)	5.85% (11/188)

Tubaki et al. 2013 [49]^‡^	RCT/randomization	606	3–84	Listhesis, disc prolapse/open instrumentation	Vancomycin powder 1 g	1.99% (6/302)	1.97% (6/304)

Abbreviations: No., number; pts, patients; RCT, randomized controlled trial; PC, prospective cohort; RC, retrospective cohort; NR, not reported. ^a^We included only deep SSI that occurred in fusion cases. Superficial SSI were excluded because the paper reports that 5 occurred in both control and treatment groups, but the paper did not discern whether these occurred in instrumented or noninstrumented cases. ^b^There is another paper by O'Neill et al. that looked at only the spine trauma cases, but Dewan et al. look at the same trauma cases plus degenerative spine disease cases. The numbers included pertain to only the degenerative spine disease cases. ^c^Sample size reflects the propensity score matched cohorts. ^g^Control group received ampicillin powder. ^‡^Only deep infections were reported in this study. ^*∗*^Studies showed a significant difference between the control and treatment groups.

**Table 2:** Summary of infection rates and methodology of studies of fracture repair.

Authors/year	Study design/method of controlling for bias	No. of pts	Age range of pts	Diagnosis	Intervention	Wound infection rates in treatment group	Wound infection rates in control group
Bibbo and Patel 2006 [50]	PC/NR	44	17–59	Calcaneal fractures	Vancomycin/DBM-calcium sulfate bone graft substitute	0% (0/33)	0% (0/11)
Cichos et al. 2021 [51]^a^	RC/multivariate analysis	789	18–89	Acetabular fractures	Vancomycin powder 1 g; Vancomycin 1 g and tobramycin 1.2 g	Vancomycin: 6.80% (20/294, 18 deep, 2 suprafascial) Vancomycin and tobramycin 9.47% (16/169, 12 deep, 4 suprafascial)	8.28% (27/326, 20 deep, 7 suprafascial)
Junker et al. 2019 [52]	PC/NR	285	18 or above	Rib fractures	Vancomycin 2 g and gentamicin 2.4 g PMMA	0% (0/8)	3.61% (10/277)
Keating et al. 1996 [53]^‡^	RC/NR	79 (79 patients, 81 fractures)	16–88	Open tibial fractures	2.4 g Tobramycin-loaded pouch	3.77% (2/55)	16.0% (4/26)
Lawing et al. 2015 [54]^*∗*^	RC/logistic regression	351	“Excluded kids <10”	Open fractures	Aminoglycosides 2 mg/mL	9.52% (16/168, 10 deep, 6 superficial)	19.67% (36/183, 26 deep, 10 superficial)
Malizos et al. 2017 [55]^*∗*^	RCT/randomization	253	20–99	Closed fractures	Antibiotic-loaded hydrogel 20–50 mg/mL	0% (0/126)	4.72% (6/127)
Moehring et al. 2000 [56]^‡,b^	RCT/randomization	55 (treatment: 22 patients, 24 fractures; Control: 33 patients, 38 fractures)	16–76	Open fractures (primarily lower extremity)	2.4 g tobramycin-impregnated beads	9.09% (2/22)	6.06% (2/33)
O'Toole et al. 2021 [57]^*∗*^^,c^	RCT/randomization	980	“Adult patients”	Tibial plateau and pilon fractures	Vancomycin powder 1 g	6.03% (29/481)	9.22% (46/499)
Ostermann et al. 1995 [58]^*∗*^	PC/NR	914 (1085 fractures)	14–99	Open fractures (primarily lower extremity)	Tobramycin-PMMA	3.67% (31/845)	12.08% (29/240)
Owen et al. 2017 [59]^‡,^^*∗*^	RC/stratification, logistic regression	140	19–65	Pelvic and acetabular fractures	Vancomycin 1 g and tobramycin 1.2 g powder	4.23% (3/71)	14.49% (10/69)
Prevost et al. 2019 [60]^‡^	RC/NR	90	NR	Open tibial fractures	Vancomycin and tobramycin powder	16.67% (11/66)	25.0% (6/24)
Qadir et al. 2020 [61]^*∗*^^,‡,d^	RC/propensity-score matching, nearest-neighbor matching	105	16–85	Bicondylar tibial plateau, tibial pilon, and calcaneus fractures	Vancomycin powder 1 g	0% (0/35)	14.29% (10/70)
Singh et al. 2015 [62]^‡^	RC/NR	93	“Adults”	Tibial plateau and pilon fractures	Vancomycin 1 g	10.00% (1/10)	16.87% (14/83)
Vaida et al. 2019 [63]^‡^	RC/NR	457	NR	Open lower extremity fractures	Vancomycin powder	8.51% (4/47)	8.78% (36/410)

Abbreviations: No., number; pts, patients; RCT, randomized controlled trial; PC, prospective cohort; RC, retrospective cohort; NR, not reported; DBM, demineralized bone matrix; PMMA, polymethyl methacrylate. ^a^We combined the two treatment groups into one intervention group in our analysis. ^b^The treatment group received just antibiotic beads, and the control group received just parenteral antibiotics. Not included are the nonrandomized third cohort that received antibiotic beads + IV. This group of patients all had Grade 3 Gustilo–Anderson open fractures. ^c^We included only deep SSI, which was the primary study outcome. Superficial SSI was excluded because the sample sizes for superficial SSI did not match those for deep SSI. ^d^This study conducted analyses using two separate methods of matching: nearest-neighbor matching and propensity score matching. It also had both prospective and retrospective control cohorts. We included the data from propensity scores matching with the prospective control cohort. ^‡^Only deep infections were reported in this study. ^*∗*^Studies showed a significant difference between the control and treatment groups.

## Data Availability

The search criteria used for article discovery are included as Supplementary Table A. All data included in this systematic review and meta-analysis were aggregated from previously published sources and have been collected from anonymous subjects.

## Supplementary Materials

Supplementary Materials
*Appendix A.* Search strategy used for Pubmed, Embase, and Web of Knowledge databases. *Appendix B.* Forest plot of pooled infection data of fracture studies with subgroup analysis by antibiotic type.
