# Supplementary material for: Prophylactic Topical Antibiotics in Fracture Repair and Spinal Fusion
Source: Adv Orthop. 2021 Oct 14;2021:1949877. doi: 10.1155/2021/1949877 (PMC8531801; doi:10.1155/2021/1949877)
Supplement: Supplementary Materials — Appendix A. Search strategy used for Pubmed, Embase, and Web of Knowledge databases. Appendix B. Forest plot of pooled infection data of fracture studies with subgroup analysis by antibiotic type. [file 1949877.f1.zip › Appendix A (1).docx]

Appendix A: Search strategy used for Pubmed, Embase, and Web of Knowledge databases

| **Pubmed** | (Local* OR intrawound* OR Local*[tiab] OR locally-applied[tiab] OR locally applied [tiab] OR “Anti-Infective Agents, Local”[Mesh] OR "Polymethyl Methacrylate"[Mesh] OR Polymethyl Methacrylate[tiab] OR PMMA[tiab] OR Bone Cement[tiab] OR Hydrogel[tiab] OR Collagen Fleece[tiab] OR "Glycopeptides"[Mesh] OR Glycopeptide*[tiab] OR impregnated[tiab] OR coated[tiab] OR topical[tiab] OR intrasite[tiab] OR intra-site[tiab] OR intra-wound[tiab] OR powder*[tiab] OR injection[tiab] OR direct*[tiab])  AND  ("Anti-Bacterial Agents" [Pharmacological Action] OR antibiotics* OR antibiotic*[tiab] OR antimicrob*[tiab] OR anti biotic*[tiab] OR anti bacterial[tiab] OR anti bacterial agents[tiab] OR antibacterial agents[tiab] OR antibacterial agents[tiab] OR bacteriocidal agents[tiab] OR bacteriocidal agents[tiab] OR bacteriocides[tiab] OR anti microb*[tiab] OR "Anti-Infective Agents"[Mesh] OR “Anti Infective”[tiab] OR “antiinfective”[tiab] OR "Microbicides"[tiab] OR "Cephalosporins"[Mesh] OR Cephalospor*[tiab] OR "Quinolones"[Mesh] OR Quinolones[tiab] OR "Rifampin"[Mesh] OR Rifampin[tiab] OR "Aminoglycosides"[Mesh] OR gentamicin[tiab] OR amikacin[tiab] OR tobramycin[tiab] OR vancomycin*[tiab] OR "Daptomycin"[Mesh] OR daptomycin*[tiab] OR "Penicillins"[Mesh] OR Penicillin*[tiab])  AND  (“fractures, bone”[MeSH Terms] OR fracture*[tiab] OR “Fracture healing”[MeSH] OR arthrodesis[MeSH Terms] OR “spinal fusion”[MeSH] OR bone fusion[tiab] OR “fracture fixation”[MeSH Terms] OR fracture fixation[tiab] OR Orthopedic trauma OR Orthopaedic trauma OR spinal surgery[tiab] OR spine surgery[tiab])  AND  ("Antibiotic Prophylaxis"[Mesh] OR prophylaxis[tiab] OR prophyla* OR prevent* OR protect*) |
| --- | --- |
| **Embase** | ('intrawound*':ab,ti OR 'local*':ab,ti OR 'locally-applied':ab,ti OR 'locally applied':ab,ti OR 'poly(methyl methacrylate)'/exp OR 'polymethyl methacrylate':ab,ti OR pmma:ab,ti OR 'palacos r':ab,ti OR 'acrylic bone cement':ab,ti OR 'hydrogel':ab,ti OR 'collagen fleece':ab,ti OR 'glycopeptide*':ab,ti OR 'bone cement':ab,ti OR 'impregnate*':ab,ti OR 'coated':ab,ti OR 'topical':ab,ti OR 'intrasite':ab,ti OR 'intra-site':ab,ti OR 'intra-wound':ab,ti OR 'powder*':ab,ti OR 'injection':ab,ti OR 'direct*':ab,ti)  AND  ('antibiotic*' OR 'antibiotic*':ab,ti OR antimicrob*:ab,ti OR 'anti biotic*':ab,ti OR 'anti microb*':ab,ti OR 'antibacterial agent*':ab,ti OR 'anti bacterial agent*':ab,ti OR 'anti infective':ab,ti OR 'antiinfective':ab,ti OR 'microbicides':ab,ti OR 'bacteriocid*':ab,ti OR 'beta lactam antibiotic'/exp OR cephalospor*:ab,ti OR 'quinolone derivative'/exp OR quinolone*:ab,ti OR 'rifampicin'/exp OR rifampin:ab,ti OR 'aminoglycoside'/exp OR gentamicin:ab,ti OR amikacin:ab,ti OR tobramycin:ab,ti OR 'vancomycin*':ab,ti OR 'daptomycin*':ab,ti OR 'penicillin derivative'/exp OR penicillin*:ab,ti)  AND  ('fracture*' OR 'fractur*':ab,ti OR 'bone fusion'/exp OR 'arthrodesis'/exp OR 'arthrodesis':ab,ti OR 'fracture fixation':ab,ti OR 'spinal fusion':ab,ti OR 'bone fusion':ab,ti OR 'orthopedic trauma':ab,ti OR 'orthopaedic trauma':ab,ti OR 'spinal surgery':ab,ti OR 'spine surgery':ab,ti)  AND  ('antibiotic prophylaxis'/exp OR 'pre emptive':ab,ti OR 'preemptive':ab,ti OR 'prophyla*':ab,ti OR 'protect*':ab,ti OR 'prevent*':ab,ti OR 'prevention'/exp OR 'protection'/exp OR 'prophylaxis'/exp OR 'prophylaxis':ab,ti OR 'infection risk’:ab,ti) |
| **Web of Knowledge** | ts=(“local*” OR “intrawound” OR “intra-wound” OR “intra wound” OR “locally-applied” OR “locally applied” OR “poly methyl methacrylate" OR "poly methylmethacrylate" OR polymethylmethacrylate OR PMMA OR "palacos R" OR "acryl bone cement" OR Hydrogel OR "collagen fleece" OR “Glycopeptide*” OR “bone cement” OR “impregnated” OR “coated” OR “topical” OR “intrasite” OR “intra-site” OR “intra-wound” OR “powder*” OR “injection” OR “direct*”)  AND  ts=(antibiotic* OR “anti biotic*” OR antimicrob* OR "anti microb*" OR “antiinfectiv*” OR  "anti infectiv*" OR antibacter* OR “anti bacter*” OR Microbicide OR bacteriocid* OR cephalospori* OR quinolo* OR rifampi* OR aminoglycosid* OR gentamycin* OR amikacin OR tobramycin OR Vancomycin OR Daptomycin OR Penicillin*)  AND  ts=("fractur*" OR "compound fractur*" OR “bone fusion” OR “arthrodesis” OR “spinal fusion” OR “bone fusion” OR “fracture fixation” OR “orthopedic trauma” OR “orthopaedic trauma” OR “fracture fixation” OR “spinal surgery” OR “spine surgery”)  AND  ts=(“antibiotic prophylaxis” OR “pre emptive” OR “preemptive” OR “prophyla*” OR “protect*” OR “prevent*” OR “infection risk” OR “risk of infection”) |
